# Identification of Defects in Low-Speed and Heavy-Load Mechanical Systems Using Multi-Fusion Analytic Mode Decomposition Method

**DOI:** 10.3390/s25061848

**Published:** 2025-03-16

**Authors:** Yanlei Liu, Kun Zhang, Miaorui Yang, Xu Zhang, Yonggang Xu

**Affiliations:** 1 Beijing Engineering Research Center of Precision Measurement Technology and Instruments, Beijing University of Technology, Beijing 100124, China; liuyl_0505@163.com (Y.L.); yangmiaorui@163.com (M.Y.); xyg_1975@163.com (Y.X.); 2 DaHe Shuzhi Information Technology Division, DaHe Shuzhi (Quanzhou) Additive Co., Ltd., Quanzhou 362000, China; xiaopangxu_zx@163.com

**Keywords:** quaternion Fourier spectrum, frequency domain fusion, analytical mode decomposition, acoustic signal processing, fault diagnosis

## Abstract

In view of the higher requirements of modern machinery for multi-sensor information acquisition and fusion technology, this paper proposes a novel multi-fusion analytic mode decomposition (MFAMD) method to separate and demodulate fault features in signals. In low-speed and heavy-load equipment, the signals collected by multiple sensors contain unknown and unequal fault features and interference. Quaternion-based frequency domain fusion technology and analytically based modal extraction technology can offer novel approaches to processing large data sets in parallel while handling lengthy signals and high sampling rates. The trend spectrum segmentation method based on quaternions optimizes the hysteresis of the binary frequency. The experimental signal verifies that the proposed method is suitable for low-speed and heavy-load bearing faults.

## 1. Introduction

Low-speed and heavy-duty equipment, which are essential parts of mechanical systems, function under more demanding circumstances than standard equipment. The components of such equipment are subjected to severe operating conditions, such as high pressure, heavy loads, high temperatures, and severe wear, over an extended period of time [1]. As the primary load-bearing elements among them, rolling bearings have a direct impact on the equipment’s overall efficiency. For this reason, it is essential from an engineering perspective to routinely diagnose rolling bearing issues in heavy-duty and low-speed equipment [2,3,4,5,6].

When it comes to diagnosing problems with mechanical equipment, traditional multi-sensor vibration acceleration acquisition methods have problems such as complex installation and difficulty in power supply. In contrast, non-contact acoustic signal detection technology has more advantages. Although signal processing is more difficult, it can effectively overcome the limitations of traditional methods. This technology was successfully used by Adam Glowacz [7] to diagnose stator faults in three-phase and induction motors in a single phase [8]. This non-contact signal processing technology has opened up a new direction for fault diagnosis research. Based on this idea, it was first used in the field of fault diagnosis by Lu [9] and Hu [10,11], who produced impressive outcomes. The method was then further extended to rotating machinery such as centrifugal pumps [12]. Tao [13] proposed a new method for adaptive separation and extraction of composite fault features of wheel hub motor bearings, which improves the accuracy and reliability of wheel hub motor bearing fault diagnosis. Feng [14] proposed a fault-tolerant collaborative control method for four-wheel drive electric vehicles, aiming to ensure the stability and safety of the vehicle through collaborative control strategies when one or more wheel hub motors fail.

High-speed bearing signals have the characteristics of short data and high information density, while low-speed and heavy-load acoustic signals face the dual challenges of large data volume and strong noise interference, which significantly increases the difficulty of fault diagnosis. Among the many diagnostic methods, wavelet transform’s superior inner product transform properties have made it a mature technology in the signal processing industry [15]. The empirical mode decomposition (EMD) method was introduced by Lei [16] and used in the diagnosis of mechanical faults. While Xue [17] innovatively applied this method to the characterization of aluminum grain size, Jiang [18] proposed a new method of center frequency mode decomposition to extract fault features and improve diagnostic accuracy. However, traditional time-domain driven signal processing methods have inherent defects such as end effects and modal aliasing [11]. To this end, researchers developed a single-channel spectrum segmentation technology based on Fourier decomposition and successfully applied it to planetary gearbox reliability detection [19]. In the field of centrifugal pump fault diagnosis, to greatly increase the effectiveness of fault identification, Kumar and Xiang [12] creatively combined symmetric single-valued neutral mutual entropy measurement with variational mode decomposition. Compared with the traditional time domain decomposition method, Chen [20] used Fourier spectrum to screen effective components in the AMD algorithm to achieve signal decomposition in different frequency bands. This method can not only effectively avoid noise and low-frequency interference through iterative calculation but also achieve accurate separation of different signal components. Subsequently, Wang [21] introduced AMD into the field of architecture, reducing the sensitivity of wavelet function parameters. Qu [22,23] proposed a multiple AMD method combining vibration response and nonlinear oscillator characteristics to determine the binary frequency from the perspective of velocity and displacement, thereby achieving accurate identification of stiffness and damping coefficients. Currently, AMD has been successfully applied in many engineering fields, such as structural inspection, wind turbine monitoring, etc. [24]. In recent years, the field of deep learning has advanced rapidly. Qu [25] proposed a method of recursive dynamic inner principal component analysis, providing significant reference for industrial automation and smart manufacturing. Zhou [26] introduced a pairwise learning framework based on prototypical representation, offering new technical support for the field of fault diagnosis. Yin [27] combined physical knowledge with data-driven methods to propose an innovative framework for predicting the remaining useful life of rolling bearings, significantly improving prediction accuracy and reliability.

Mechanical equipment is irregular in operation and usually contains multiple components, such as component vibration, modulation signals, material impact noise, and strong environmental noise. The efficiency of the AMD method is affected by noise interference, etc. The traditional method requires manual observation to determine the key frequencies. Among them, the number and position of the bisection frequencies directly affect the diagnostic accuracy. The frequency between the adjacent frequencies is selected as the bisection frequency. The spectrum is divided into several unique frequency bands using iterative processes by the bisection frequency, each of which is linked to a specific diagnostic result [28]. To make the bisection frequency selection process easier, Wang [29] replaced the conventional Fourier spectrum with the autoregressive power spectrum in a creative way, which significantly improved the practicality of the method. Zhao [30] achieved accurate determination of the optimal cutoff frequency by combining the algorithm with calculation. However, there is still room for improvement in existing methods, such as by addressing problems with large data volumes and adaptive spectrum segmentation. In response to these challenges, the dual-channel acoustic signal processing method developed by Bendoumia [31] has made breakthrough progress. At the same time, Jeon [32] optimized vibration positioning and production control using a multi-channel distributed speaker system. Hamdan [33] constructed a multi-channel crosstalk elimination system based on singular value decomposition, laying the foundation for subsequent research. With the advancement of technology, more innovative signal fusion methods have emerged: Salah [34] implemented audio watermarking using quaternion discrete Fourier transform, while Martins [35] successfully solved the rotation lock problem of curvature parameters of avionics equipment through the quaternion method. Jiang [36] proposed a semi-supervised optimized temperature-guided PCL framework based on multi-sensor data fusion for mechanical equipment fault diagnosis, aiming to improve diagnostic accuracy and reliability. Yi [37] innovatively used phase space reconstruction technology to convert four-channel signals into Hankel matrices and then constructed quaternion trajectory matrices, successfully realizing accurate fault diagnosis. On this basis, Ma [38] combined symplectic geometry, singular spectrum analysis (SSA), and quaternion theory to effectively perform fault diagnosis. These studies show that quaternion methods play a key role in multi-channel signal synchronization processing and provide new solutions for fault diagnosis of complex mechanical systems. Multichannel signal processing significantly enhances the accuracy and reliability of signal analysis by simultaneously acquiring and analyzing signals from multiple sensors. Through the fusion processing of multichannel signals, it can effectively suppress noise interference in individual channels, improve the signal-to-noise ratio (SNR), and thereby extract fault features more clearly. Additionally, multichannel signal processing enables the extraction of higher-dimensional features, providing richer feature information for fault diagnosis and improving diagnostic accuracy.

When working with heavier loads and lower speeds, the conventional singular spectrum analysis (SSA) method suffers from low matrix construction efficiency. This paper proposes a multi-fusion analysis mode decomposition (MFAMD) method to process multi-channel acoustic signals of more complex mechanical systems to achieve efficient extraction of fault information. By effectively extracting fault information from signals, the MFAMD approach can contribute to the advancement of mechanical fault diagnosis. The MFAMD method is proposed in this paper, which offers a new direction for processing multi-channel signals by fusing multiple groups or multi-channel experimental data in the frequency domain. The suggested MFAMD technique can adaptively divide frequency bands, diagnose faults, and fuse multi-channel signals in the frequency domain. Under complex operating conditions, MFAMD demonstrates superior adaptability to the nonlinear and non-stationary characteristics of signals, thereby enhancing the reliability of analysis. The method’s ability to detect fault information in acoustic signals and carry out fault diagnosis is demonstrated by simulation and experimental results. This paper has the following structure: In particular, Section 2 presents the MFAMD method and multi-channel signal frequency domain fusion technology; Section 3 confirms the method’s efficacy using simulation signals; and the technique is effective in locating flaws in the bearing’s inner and outer rings.

## 2. Theoretical Descriptions

**Figure 1:**
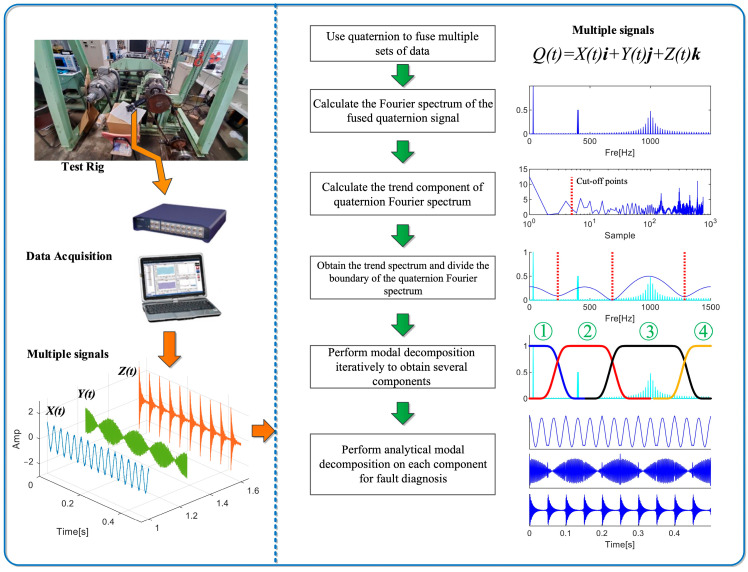
The flowchart of MFAMD.

The frequency domain fusion of multi-channel acoustic signals is creatively realized by the multi-fusion analytical modal decomposition method presented in this paper, yielding more suitable frequency band components, and finally performs fault diagnosis. Firstly, a quaternion-based multi-channel signal frequency domain fusion technology is proposed to achieve accurate analysis of multiple groups or multi-channel signals; secondly, a quaternion Fourier spectrum trend spectrum segmentation technology is proposed to realize the adaptive and accurate capture of binary frequency. The precision and effectiveness of mechanical fault diagnosis in challenging operating environments have been greatly enhanced by these developments. As shown in Figure 1, the MFAMD method flow chart, the specific steps are as follows:

(1) Synchronously collect multi-channel signals.
(2) Use quaternion theory to fuse multiple channels or multiple groups of signals into quaternion signals.
(3) Calculate the spectrum of multiple groups or multi-channel signals after fusion in the frequency domain.
(4) Acquire the trend spectrum and apply the Fourier transform to the fused spectrum.
(5) Obtain multiple frequency band components and split the fused spectrum into frequency bands using the trend spectrum.
(6) Perform model decomposition iteratively to obtain several components.

### 2.1. Multi-Signal Frequency Domain Fusion Method

Multi-channel data synchronous and asynchronous acquisition technologies have advanced dramatically with the development of sensor technology. The system can store numerous signals for a long time because of the advancement in big data storage technologies. At the same time, the improvement of the collector’s sensitivity allows the signal to contain richer detailed information, which may be valuable fault characteristics or interference noise. Especially under low speed and heavy load conditions, weak fault signals often have long periodic characteristics, which hide key vibration details. Thus, the effective extraction of weak fault information is aided by the use of multi-data or multi-sensor signal fusion technology. This paper efficiently fuses multiple sets of data signals using the quaternion method.

The theory of quaternions, which are made up of three imaginary parts and one real part, was put forth by Hamiltonian [39], forming a unique mathematical structure. Quaternions belong to the hypercomplex number group, and their mathematical expression can be described as follows [40]:

(1) Q=a+bi+cj+dk∈H,

where i,j,k are imaginary units; a, b, c, d∈R are real numbers; and they satisfy the following special properties, especially the unique multiplication rules:

(2) $$i^{2}=j^{2}=k^{2}=ijk=-1,$$

(3) ij=−ji=k,jk=−kj=i,ki=−ik=j,

Due to the non-commutativity of quaternion multiplication, its computational complexity increases significantly. This paper presents Ell’s quaternion Fourier transform (QFT) method, which effectively fuses quaternion signals [41]. QFT can be considered as an extension of Fourier transform; QFT is particularly suitable for processing signals and images composed of three-dimensional or four-dimensional samples. For a quaternion numerical signal f(t), its one-dimensional right-hand QFT is defined as follows:

(4) $$F^{R}\left(\omega \right)=\kappa_{1}\int_{-\infty }^{\infty }f(t)e^{\mp \mu \omega t}dt,$$

where −μ represents the forward transformation, and +μ represents the reverse transformation, which is essentially different from the inverse transformation given by the following equation:

(5) $$f\left(t\right)=\kappa_{2}\int_{-\infty }^{\infty }F^{R}\left(\omega \right)e^{\pm \mu \omega t}d\omega ,$$

where $F^{R}\left(\omega \right)$ represents the spectrum of the quaternion numerical function $f\left(t\right)$. A normalized quaternion, denoted by ***μ***, is also known as the quaternion’s rotation axis.

(6) $$\mu =\frac{a}{\sqrt{a^{2}+b^{2}+c^{2}}}i+\frac{b}{\sqrt{a^{2}+b^{2}+c^{2}}}j+\frac{c}{\sqrt{a^{2}+b^{2}+c^{2}}}k,$$

The scaling coefficients satisfy $\kappa_{1}\kappa_{2}={\left(2\pi \right)}^{-1}$. When $\kappa_{1}=\kappa_{2}$, the transformation constitutes a unitary transformation. For the quaternion numerical signal $f\left(t\right)$, its one-dimensional left-hand QFT and its inverse transformation can be expressed as follows:

(7) $$F^{L}\left(\omega \right)=\kappa_{-}\int_{-\infty }^{\infty }e^{\mp \mu \omega t}f(t)dt,$$

(8) $$f\left(t\right)=\kappa_{+}\int_{-\infty }^{\infty }e^{\pm \mu \omega t}F^{L}\left(\omega \right)d\omega$$

Due to the unique characteristics of quaternions, Sangwine [42] developed the discrete quaternion Fourier transform (DQFT), which was utilized in the field of color image processing to resolve the problem between the imaginary parts of the signal. However, due to the non-commutativity of quaternion multiplication, direct calculation using the DQFT formula is computationally demanding and inefficient. In order to achieve this, researchers developed the quaternion fast Fourier transform (QFFT) [43], which breaks down the original data and uses the fast Fourier transform algorithm for computation, greatly increasing computational efficiency.

Assuming a quaternion in a standard basis, {1,i,j,k} is expressed as q=a+bi+cj+dk, and a new basis $B=\{1,\mu_{1},\mu_{2},\mu_{1}\mu_{2}\}$ can be constructed, which is expressed as follows:

(9) $$B=\left(\begin{array}{lll} \mu_{1i} & \mu_{1j} & \mu_{1k}\\ \mu_{2i} & \mu_{2j} & \mu_{2k}\\ \mu_{3i} & \mu_{3j} & \mu_{3k} \end{array}\right)$$

In the new basis representation, the four numerical components of the quaternion are as follows:

(10) $$\left\{\begin{array}{c} a^{\prime }=a\\ b^{\prime }=\langle a+bi+cj+dk,\mu_{1}\rangle \\ c^{\prime }=\langle a+bi+cj+dk,\;\mu_{2}\rangle \\ d^{\prime }=\langle a+bi+cj+dk,\mu_{3}\rangle \end{array}\right.$$

where $\mu_{3}=\mu_{1}\mu_{2}=V\left(\mu_{1}\right)\times V\left(\mu_{2}\right)$ and $\langle \cdot \rangle$ represents the scalar product.

After basis transformation, the quaternion signal can be decomposed into two complex signals:

(11) $$\left\{\begin{array}{c} z_{1}=a^{\prime }+b^{\prime }I\\ z_{2}=c^{\prime }+d^{\prime }I \end{array}\right.$$

where I stands for −1’s complex root. QFT can be shown in a different way:

$$F\left(u\right)=\sum_{n=1}^{N}e^{-\mu 2\pi \frac{nu}{N}}\left(a^{\prime }+b^{\prime }\cdot \mu_{1}+c^{\prime }\cdot \mu_{2}+d^{\prime }\cdot \mu_{3}\right)$$

(12) $$=\sum_{n=1}^{N}e^{-\mu 2\pi \frac{nu}{N}}\left(a^{\prime }+b^{\prime }\mu_{1}\right)+\sum_{n=1}^{N}e^{-\mu 2\pi \frac{nu}{N}}\left(c^{\prime }+d^{\prime }\mu_{1}\right)\mu_{2}$$

The secret to this part is combining the frequency domain of several sets of related signals or multi-channel signals. The frequency domain of multiple groups of signals is fused, and the fused spectrum is obtained. The objective function of the spectrum calculation is more concise and effective. To demonstrate the method of multi-signal frequency domain fusion, this section constructs a three-channel simulation signal: the X channel contains an interference signal with modulation information, and the Z channel signal also contains modulation information, with center frequencies of 1000 Hz and 4000 Hz respectively; with a center frequency of 2500 Hz, the Y channel is a recurring pulse signal that simulates an outer ring fault in the bearing.

(13) $$\left\{\begin{array}{c} X\left(t\right)=2\sin\left(100\pi t\right)\times \sin\left(2000\pi t+\sin\left(200\pi t\right)\right)\\ Y\left(t\right)=\sum_{i=1}^{M}6e^{-2g\pi f_{n}^{i}t}\times \sin\left(2\pi f_{n}^{i}t\times \sqrt{1-g^{2}}\right)\\ Z\left(t\right)=2\sin\left(100\pi t\right)\times \sin\left(8000\pi t+\sin\left(200\pi t\right)\right) \end{array}\right.$$

where coefficient of damping g=0.07, M=10, and the fault frequency is 100 Hz. An amount of 10 dB Gaussian white noise is added to all three channels.

**Figure 2:**
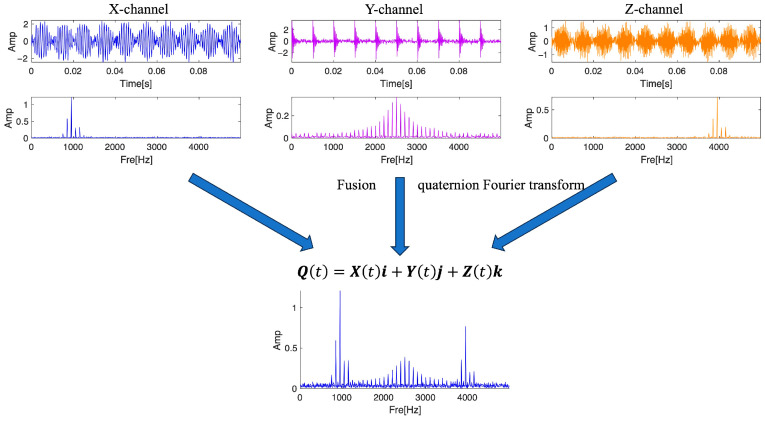
Schematic diagram of multi-signal fusion method based on quaternions.

In the frequency domain, the three channel signals’ center frequencies have varying values, which can more clearly illustrate QFT’s benefits. As shown in Figure 2, The efficacy of this technique is demonstrated by the spectrum of multi-channel signal fusion, which preserves certain features of each channel signal in the frequency domain.

### 2.2. Spectrum Segmentation Method Based on Multi-Signal Frequency Domain Fusion

Due to problems that traditional time domain decomposition technology commonly faces, such as mode aliasing and boundary effects, the frequency domain decomposition method has received a lot of attention and development in recent years. This study builds the mathematical expression of the quaternion Fourier transform $\hat{q}\left(\omega \right)$ of the signal $q\left(t\right)$ as follows and, based on the existing accomplishments, presents the quaternion Fourier spectrum trend analysis method for spectrum segmentation:

(14) $$\hat{q}\left(\omega \right)=\int_{-\infty }^{+\infty }q\left(t\right)e^{-i\omega t}dt$$

The spectrum $\left|\hat{q}\left(\omega \right)\right|$ containing only the real part is discretized, where $v\left(n\right)=\left|\hat{q}\left(n\right)\right|$, =1,2,3,…, L. L is the length of $v\left(n\right)$. $v\left(n\right)$ is a set of non-negative sequences. The Fourier transform is then discretized:

(15) $$\hat{v}\left(u\right)=\sum_{n=1}^{L}v\left(n\right)e^{-i\frac{2\pi }{L}un}$$

Similar to the Fourier transform, $\hat{v}\left(u\right)$ consists of a real part and an imaginary part. In the initial decomposition, a small part of $\hat{v}\left(u\right)$ can be selected for inverse transformation, and the initial intercept length can be assumed to be 5, which can be set based on experience. The selection of the intercept length is a critical step that directly impacts the performance of the algorithm and the accuracy of the results. Choosing either an excessively long or short truncation length can adversely affect the effectiveness of spectrum segmentation. In order to optimize the computational efficiency, it is recommended that the reader can dynamically adjust this parameter based on the number of extreme points, thereby effectively reducing the iteration time. It should be mentioned that if the initial intercept length is set too small, the spectrum may not be precisely divided by the trend component. This is primarily because there is not the required minimum point. The trend component can be written as follows if ${\hat{v}}_{k}(u)$ is the chosen portion of $\hat{v}\left(u\right)$:

(16) $$T_{k}\left(f\right)=\left|\int_{-\infty }^{+\infty }{\hat{v}}_{k}(u)e^{i2\pi fu}du\right|$$

where the first 5×k points of $\hat{v}\left(u\right)$ are denoted by ${\hat{v}}_{k}(u)$, while $T_{k}\left(f\right)$ stands for the degradation layer’s constituents.

**Figure 3:**
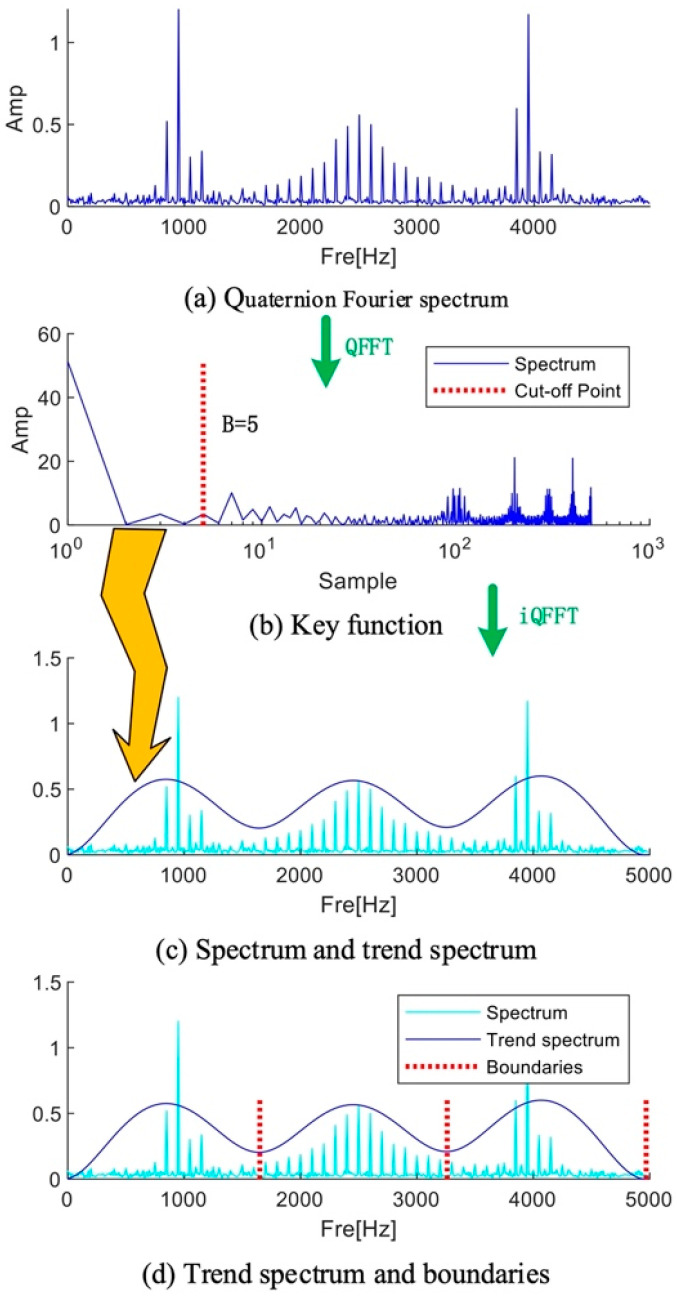
Flowchart of calculating trend spectrum and boundaries.

Figure 3 shows the process of trend spectrum calculation and boundary division. The value of parameter B needs to be set based on experience. Studies have shown that the increase in the B value will lead to an increase in the complexity of the trend spectrum and an increase in the number of boundaries; conversely, when the B value decreases, the trend spectrum presents a smoothing feature, and the number of boundaries decreases accordingly. It is worth noting that all the divided boundaries correspond to equally divided frequency points.

### 2.3. Multi-Fusion Analytic Mode Decomposition

This study suggested a multi-channel signal frequency domain fusion method to address the shortcomings of the AMD method. It combines quaternion Fourier spectrum trend analysis with spectrum segmentation to achieve precise bisection frequency estimation. Let $g\left(t\right)$ be the signal in the time domain. Since the original signal may have been composed of several different parts, it can be expressed as follows:

(17) $$g\left(t\right)=\sum_{i=1}^{M}g_{i}(t)$$

where the number of components is M; i=1,2,3…M.

If the quaternion Fourier spectrum $\hat{G}(\omega )$ of the signal and its components exists, then the frequency ${\hat{G}}_{M}(\omega )$ of $g_{i}(t)$ can be defined as important frequencies. The bisecting frequency can be obtained from the important frequency, expressed as $\omega_{b}$: $\omega_{b0}=0$ Hz, $\omega_{bM}=\pi$. The bisecting frequencies obtained by the method proposed in Section 2.2 can be expressed as $\sum_{i=0}^{M}\omega_{bi}$. Center frequencies are regarded as crucial frequencies. $\omega_{\mathrm{ci}}$ is the interval’s center frequency $\left[\omega_{b(i-1)},\;\omega_{bi}\right]$, and $∆\omega_{i}=\omega_{bi}-\omega_{b(i-1)}$ is the bandwidth. ${\hat{G}}_{i}\left(f;\omega_{ci},∆\omega_{i}\right)$ in the time domain, and $g_{i}\left(t;\omega_{ci},∆\omega_{i}\right)$ in the frequency domain can be used to represent the signals in the interval. Consequently, Equation (17) can be reformulated as follows:

(18) $$g\left(t\right)=\sum_{i=1}^{M}g_{i}\left(t;\omega_{ci},∆\omega_{i}\right)$$

One definition of the bisection frequency is the boundary:

(19) $$\left\{\begin{array}{c} \omega_{b0}<\left|\omega_{c1}\right|<\omega_{b1}\\ \omega_{b1}<\left|\omega_{c2}\right|<\omega_{b2}\\ \omega_{b2}<\left|\omega_{c3}\right|<\omega_{b3}\\ \ldots \\ \omega_{b\left(M-1\right)}<\left|\omega_{cM}\right|<\omega_{bM} \end{array}\right.$$

When the first component is extracted, assume that $s_{k}(t)$ represents a trigonometric function. When k=m, $s_{k}\left(t\right)=s_{m}\left(t\right)=\cos\left(\omega_{b1}t\right);$ when k=n, ${s_{k}\left(t\right)=s}_{n}\left(t\right)=\sin(\omega_{b1}t)$. The Hilbert transform (H [·]) of $s_{k}(t)g(t)$ can be expressed as follows:

(20) $$H[s_{k}(t)g(t)]=H[s_{k}(t)g_{1}\left(t\right)]+H[s_{k}(t)g_{Res1}\left(t\right)]$$

Formula (20) can be expressed in another form:

(21) $$H[s_{k}(t)g(t)]=g_{1}\left(t\right)H[s_{k}(t)]+s_{k}(t)H[g_{Res1}\left(t\right)]$$

Substitute c and s into Equation (21) respectively to get the following:

(22) $$\left\{\begin{array}{l} H\left[s_{m}\left(t\right)g\left(t\right)\right]=g_{1}\left(t\right)H\left[s_{m}\left(t\right)\right]+s_{c}\left(t\right)H\left[g_{Res1}\left(t\right)\right]\\ H[s_{n}(t)g(t)]=g_{1}\left(t\right)H[s_{n}(t)]+s_{n}(t)H[g_{Res1}\left(t\right)] \end{array}\right.$$

Then,

(23) $$\left\{\begin{array}{l} H\left[g_{Res1}\left(t\right)\right]=\frac{H\left[s_{m}\left(t\right)g\left(t\right)\right]-g_{1}\left(t\right)H\left[s_{m}\left(t\right)\right]}{s_{m}\left(t\right)}\\ H[s_{n}(t)g(t)]=g_{1}\left(t\right)H[s_{n}(t)]+s_{n}(t)\frac{H\left[s_{m}\left(t\right)g\left(t\right)\right]-g_{1}\left(t\right)H\left[s_{m}\left(t\right)\right]}{s_{m}\left(t\right)} \end{array}\right.$$

Thus, such results can be obtained:

(24) $$\left\{\begin{array}{c} g_{1}\left(t\right)=\frac{s_{n}\left(t\right)H\left[s_{m}\left(t\right)g\left(t\right)\right]-s_{m}\left(t\right)H\left[s_{n}\left(t\right)g\left(t\right)\right]}{s_{n}\left(t\right)H\left[s_{m}\left(t\right)\right]-s_{m}\left(t\right)H\left[s_{n}\left(t\right)\right]}\\ H[g_{Res1}\left(t\right)]=\frac{H\left[s_{m}\left(t\right)\right]H\left[s_{n}\left(t\right)g\left(t\right)\right]-H\left[s_{n}\left(t\right)\right]H\left[s_{m}\left(t\right)g\left(t\right)\right]}{s_{n}\left(t\right)H\left[s_{m}\left(t\right)\right]-s_{m}\left(t\right)H\left[s_{n}\left(t\right)\right]} \end{array}\right.$$

Given that $s_{n}\left(t\right)$ has the Hilbert transform $H\left[s_{n}\left(t\right)\right]=-\cos(\omega_{b}t)$, $s_{m}\left(t\right)$ has the Hilbert transform $H\left[s_{m}\left(t\right)\right]=\sin(\omega_{b}t)$, and $s_{n}\left(t\right)H\left[s_{m}\left(t\right)\right]-s_{m}\left(t\right)H\left[s_{n}\left(t\right)\right]=1$. Consequently, Equation (24) can be reduced to the following:

(25) $$\left\{\begin{array}{c} g_{1}\left(t\right)=s_{n}\left(t\right)H\left[s_{m}\left(t\right)g\left(t\right)\right]-s_{m}\left(t\right)H\left[s_{n}\left(t\right)g\left(t\right)\right]\\ H\left[g_{Res1}\left(t\right)\right]=H\left[s_{m}\left(t\right)\right]H\left[s_{n}\left(t\right)g\left(t\right)\right]-H\left[s_{n}\left(t\right)\right]H\left[s_{m}\left(t\right)g\left(t\right)\right] \end{array}\right.$$

Therefore, the first component can be extracted through $\omega_{b1}$:

(26) $$\left\{\begin{array}{cc} g_{1}\left(t\right) & =\sin\left(\omega_{b1}t\right)H\left[\cos\left(\omega_{b1}t\right)\left[g\left(t\right)\right]\right]\\ & -\cos\left(\omega_{b1}t\right)H\left[\sin\left(\omega_{b1}t\right)\left[g\left(t\right)\right]\right]\\ g_{Res1}\left(t\right) & =g\left(t\right)-g_{1}\left(t\right) \end{array}\right.$$

where $g_{Res1}\left(t\right)$ is the high-frequency residual component. The second low-frequency component can be extracted by $\omega_{b2}$:

(27) $$\left\{\begin{array}{cc} g_{2}\left(t\right) & =\sin\left(\omega_{b2}t\right)H\left[\cos\left(\omega_{b2}t\right)\left[g_{Res1}\left(t\right)\right]\right]\\ & -\cos\left(\omega_{b2}t\right)H\left[\sin\left(\omega_{b2}t\right)\left[g_{Res1}\left(t\right)\right]\right]\\ g_{Res2}\left(t\right) & =g_{Res1}\left(t\right)-g_{2}\left(t\right) \end{array}\right.$$

By analogy, we can get the following:

(28) $$\left\{\begin{array}{cc} g_{i}\left(t\right) & =\sin\left(\omega_{bi}t\right)H\left[\cos\left(\omega_{bi}t\right)\left[g_{Res\left(i-1\right)}\left(t\right)\right]\right]\\ & -\cos\left(\omega_{bi}t\right)H\left[\sin\left(\omega_{bi}t\right)\left[g_{Res(i-1)}\left(t\right)\right]\right]\\ g_{Resi}\left(t\right) & =g_{Res(i-1)}\left(t\right)-g_{i}\left(t\right) \end{array}\right.$$

The original signal can therefore be represented by several components and a residual component.

One residual component and the sum of multiple components can be used to represent the original signal:

(29) $$g\left(t\right)=\sum_{i=1}^{M-1}g_{i}\left(t\right)+g_{Res(M-1)}\left(t\right)$$

where another way to think of $g_{Res(M-1)}\left(t\right)$ is the last element that has the highest frequency, which may be regarded as the final element with the highest frequency: $G_{M}$.

**Figure 4:**
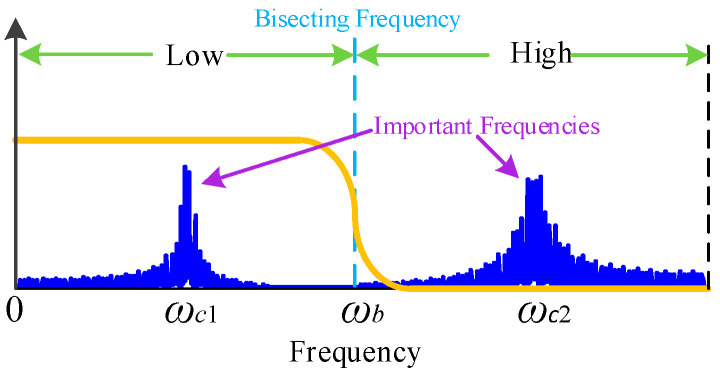
Bisecting frequency.

The signal has two important components that are easily distinguished in the spectral domain, as seen in Figure 4. The key frequencies are cut in half to successfully separate the two components. The low-frequency component is first extracted by signal processing, and the high-frequency residual that is left over can be the object of further analysis.

**Figure 5:**
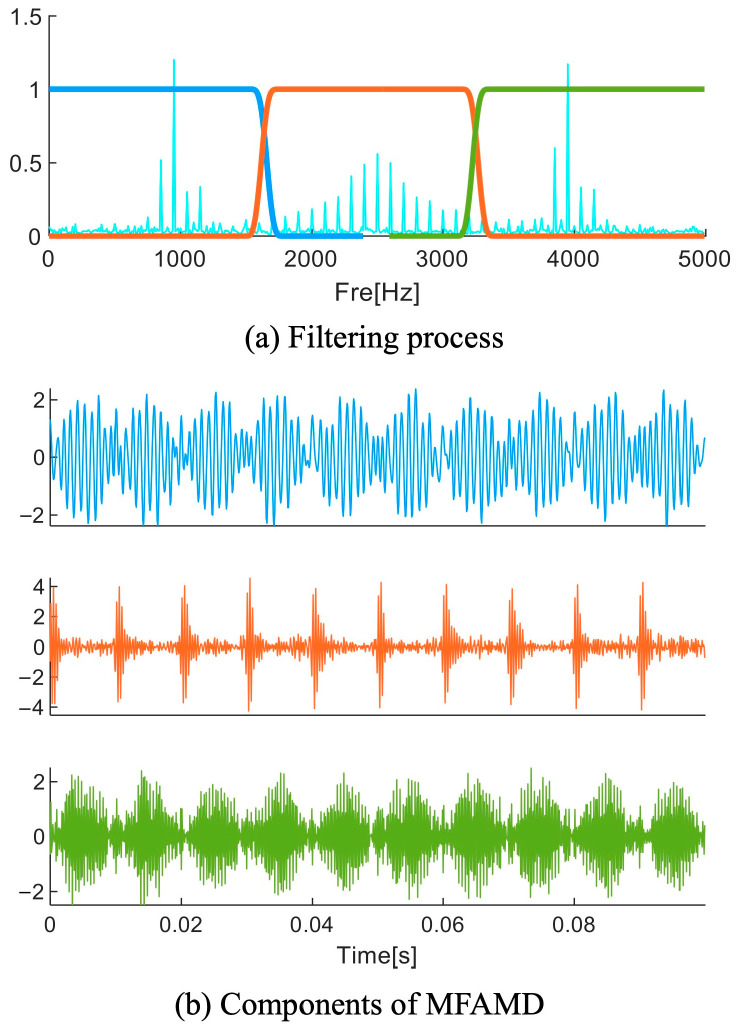
The MFAMD filtering procedure and outcomes.

Based on the bisection frequency obtained in Section 2.2, this section adopts an iterative decomposition strategy. This method continuously filters out low-frequency components, making the proposed signal separation scheme functionally similar to a set of filters. Figure 5 shows the filtering process and its processing results in detail.

## 3. Simulation Verification Analysis

**Figure 6:**
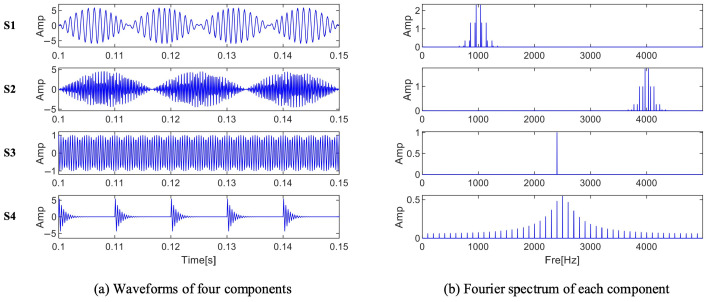
Signal components and spectrum of three-channel signals.

Equation (30) illustrates how a set of three-channel simulation verification signals are simulated in this section, which simulates the low-frequency and high-frequency information modulation information that will appear in the normal operation of the bearing, as well as other interference information and noise. As shown in Figure 6a, the amplitudes of the signals of each component are not consistent center frequencies. The Fourier spectrum of the four distinct components is shown in Figure 6b. The figure shows the details of two modulation signals, periodic pulses and harmonic signals, with center frequencies of 1000 Hz, 4000 Hz, 2400 Hz, and 2500 Hz, respectively. The viability and efficiency of the approach suggested in the article are illustrated below via the processing of the simulation signal.

(30) $$\left\{\begin{array}{c} s_{X}=6\sin\left(80\pi t\right)\times \sin\left(2000\pi t+\sin\left(200\pi t\right)\right)\\ +\sum_{i=1}^{M}6e^{-2g\pi f_{n}^{i}t}\times \sin\left(2\pi f_{n}^{i}t\times \sqrt{1-g^{2}}\right)\\ s_{Y}=4.5\sin\left(80\pi t\right)\times \sin\left(8000\pi t+\sin\left(200\pi t\right)\right)\\ +\sum_{i=1}^{M}6e^{-2g\pi f_{n}^{i}t}\times \sin\left(2\pi f_{n}^{i}t\times \sqrt{1-g^{2}}\right)\\ s_{Z}=2\sin\left(80\pi t\right)\times \sin\left(2000\pi t+\sin\left(200\pi t\right)\right)\\ +\cos\left(4800\pi t\right) \end{array}\right.$$

where the damping coefficient is 0.07, the natural frequency is 2400 Hz, and the fault characteristic frequency is 100 Hz. To more realistically simulate actual experimental data, Gaussian white noise with a signal-to-noise ratio of −2 dB was added to each of the three-channel signals.

Due to the different fault locations and sensor placements, the signals collected by the three channels are not exactly the same. There may be a lot of fault information in some of the signals that certain channels collect, while some of the signals collected by some channels may contain less or almost no fault information. Therefore, this section considers these effects and randomly constructs the following simulation signals, where the Z channel only contains interference information, while the X and Y channels primarily contain a lot of modulation information and a little bit of fault information.

**Figure 7:**
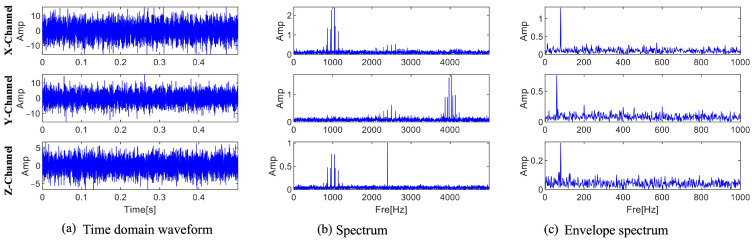
Time domain waveform, spectrum, and envelope spectrum of the three-channel signal.

**Figure 8:**
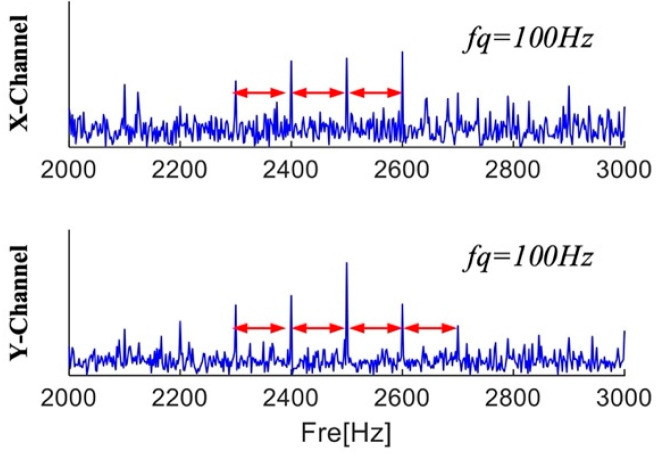
Magnified view of the X and Y channels.

The location of the fault information cannot be ascertained because, as Figure 7 illustrates, the Fourier spectra of the three channels only show three ranges with center frequencies and sidebands. According to Equation (30), the center frequency, sideband frequency, and characteristic frequency of the fault information are 2500 Hz, 100 Hz, and 100 Hz, respectively. Figure 7c displays the envelope spectra of the three channel signals. The characteristic frequencies obtained by the three channels are different from the fault characteristic frequencies. Interference information and noise drown out the fault information, and no fault frequency multiplication is found in the envelope spectrum. A close-up of the X and Y channel spectra is displayed in Figure 8. There are multiple frequencies with a 100 Hz interval that contain fault information in the local enlarged view of the two channels. Dividing the frequency bands can separate the fault information from interference and noise, and the envelope spectrum separated in each frequency band can be calculated to diagnose faults.

**Figure 9:**
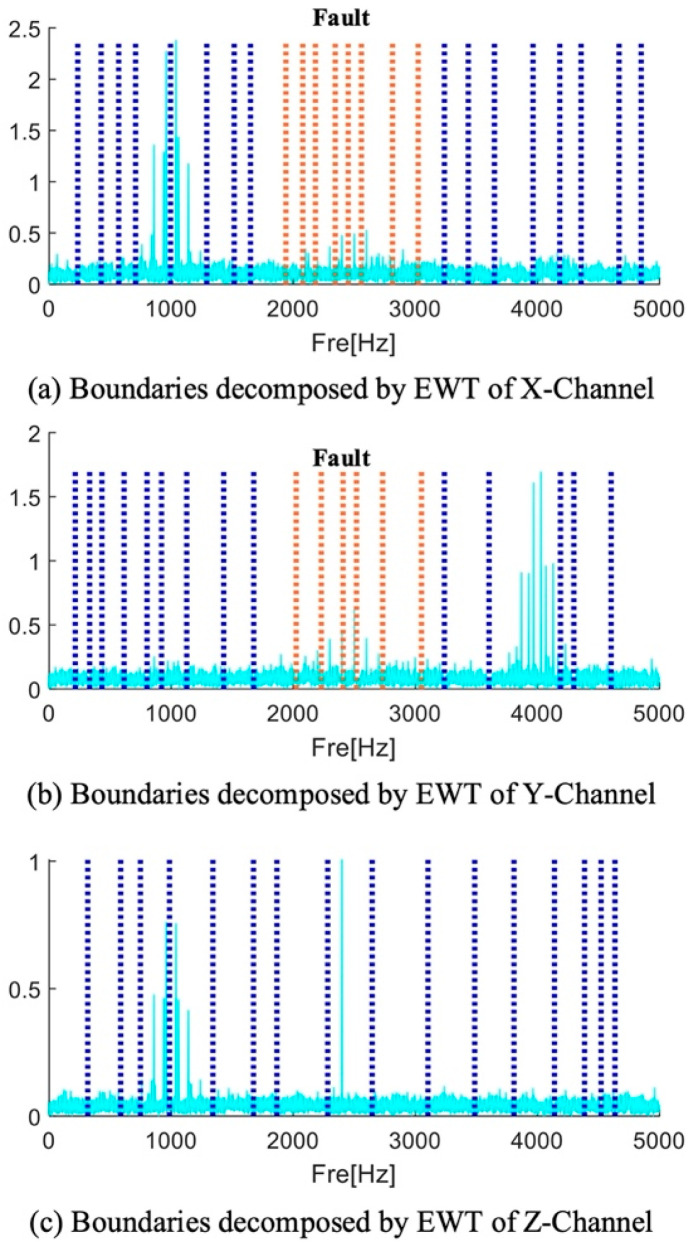
EWT divides the frequency band of each channel signal.

Gilles [44] proposed a scale space representation-based adaptive empirical segmentation method that is frequently applied in fault diagnosis. Thus, by contrasting the suggested approach with superior spectrum segmentation, its benefits are illustrated. The boundary distribution of the signals of the three channels after EWT processing is shown in Figure 9. These boundary distributions are shown to be extremely tight. The EWT technique is unable to fully extract the frequency band containing the fault information around the 2500 Hz range. The frequency band containing fault information is divided by the orange boundary, as shown in Figure 9. Because there is less fault information in these divided frequency band components, the fault frequency multiplication cannot be seen in the frequency band components’ envelope spectrum. Therefore, more research is required to determine the best way to divide the boundary. Figure 9 illustrates the results of applying the EWT spectrum segmentation method to a three-channel signal. The blue dashed lines in the figure represent the results obtained by processing the channel signals using the EWT method. The orange dashed lines indicate the segmentation boundaries at the frequency bands where fault information is located, aiming to demonstrate that the EWT method fails to effectively extract the fault information during spectrum segmentation.

**Figure 10:**
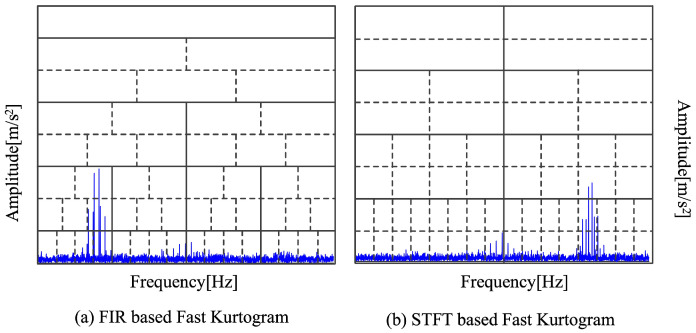
Two segmentation framework diagrams of fast kurtogram.

Antoni [45] suggested another traditional spectrum segmentation technique. There are two segmentation techniques in the fast kurtosis (FK). A schematic diagram of the two segmentation techniques is displayed in Figure 10. The graphic illustrates how the frequency band holding fault information is not always kept in each decomposition stage. The fault frequency band becomes fully segmented as the number of breakdown layers rises, which influences the subsequent fault identification step. Furthermore, neither of the segmentation frameworks can be adjusted adaptively to accommodate spectrum fluctuations because they are fixed. The previously described fault frequency band will be split into several sections since it is clear from Figure 10 that the center line of the FK segmentation framework is precisely aligned with the center frequency of 2500 Hz.

**Figure 11:**
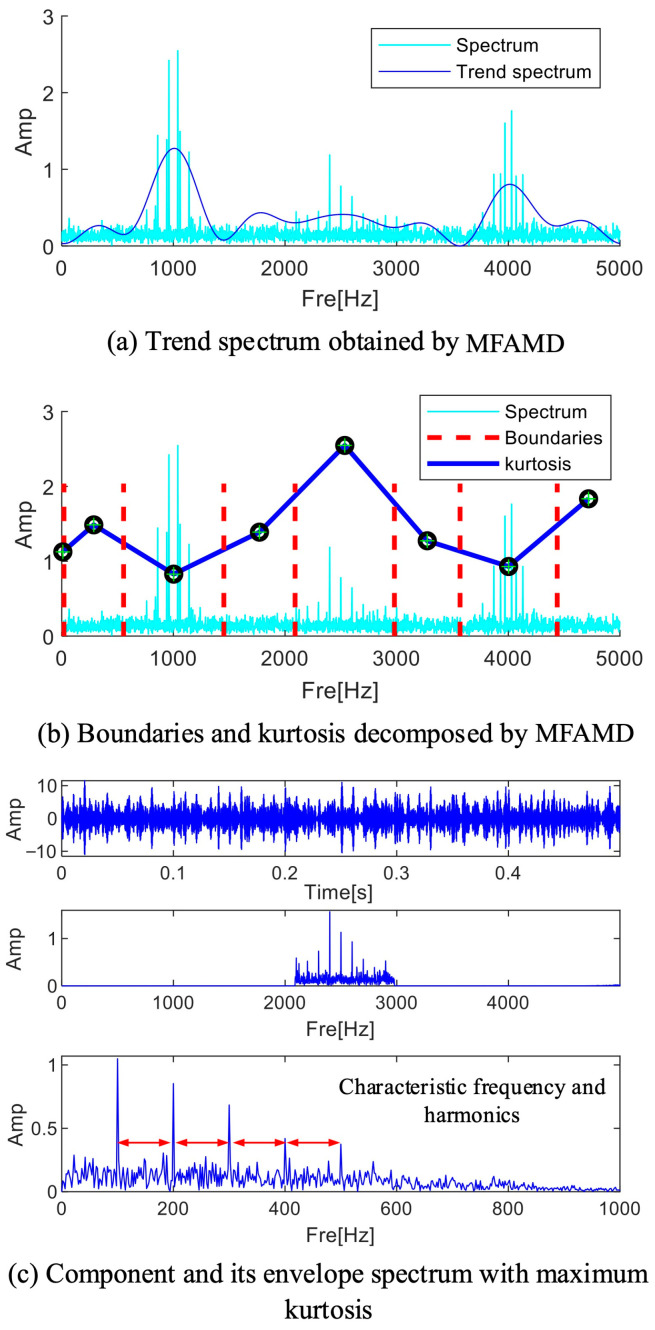
The results processed by MFAMD.

A trend spectrum resembling the spectrum fluctuation trend following multi-channel signal fusion can be obtained by processing the signal using the MFAMD method suggested in this paper, as illustrated in Figure 11a. As seen in Figure 11a, the trend spectrum fluctuates more smoothly and has fewer extreme points. As seen in Figure 11b, the MFAMD method splits the signal into eight sections, with the frequency range [2000 Hz–3000 Hz] storing nearly all of the fault information. Each frequency band component’s kurtosis index can be determined by reconstructing its time domain waveform. As shown in Figure 11b, the frequency band component here contains more fault information when the kurtosis index value is the highest. The kurtosis index is highly sensitive to fault information; therefore, after completing the frequency band division, the kurtosis index is employed to quantify the fault information. To reconstruct the signal, the fifth frequency band component in Figure 11b with the highest kurtosis value is chosen, and Figure 11c displays the results of the signal’s and the envelope’s spectrum calculations. The effectiveness of the technique is demonstrated by the reconstructed envelope spectrum, which clearly displays fault frequency multiples and shows that the suggested strategy can preserve the frequency range that contains the fault information.

**Figure 12:**
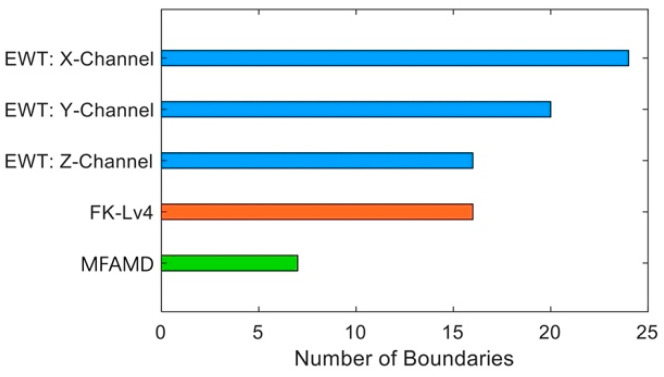
Boundary quantity comparison chart.

The number of boundaries produced by the three approaches is statistically displayed in Figure 12 to illustrate the benefit of the MFAMD method in boundary division. The figure illustrates how the MFAMD approach is more likely to preserve the fault frequency band and yields fewer boundaries. The segmentation boundary comparisons between the two methods in Figure 9 and Figure 11 demonstrate that the MFAMD method yields a substantially lower number of boundaries. While the EWT approach splits the fault information into many sections, the fifth frequency band component of the MFAMD method preserves the entire frequency band carrying the fault information. The benefits of the proposed MFAMD method are extensively illustrated in this section.

The algorithm’s execution speed is typically directly correlated with the number of boundaries. The results of the fault diagnosis are somewhat influenced by the quantity and location of effective boundaries. In this section, the fault center frequency of the simulation signal is designed to be 2500 Hz, and [2000–3000 Hz] is taken as the optimal decomposition frequency band. The number of boundaries and effective sidebands that the three approaches produced in the [2000–3000 Hz] frequency range are displayed in Table 1. The MFAMD approach yields the fewest boundaries, demonstrating that the suggested approach better preserves the fault frequency band and confirming its efficacy.

**Figure 13:**
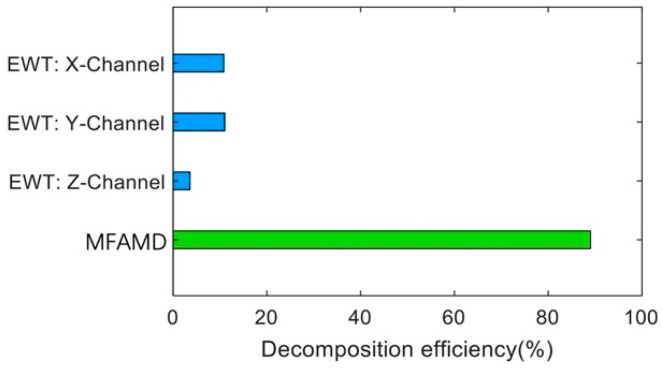
Comparison of decomposition efficiency between EWT and MFAMD.

**Figure 14:**
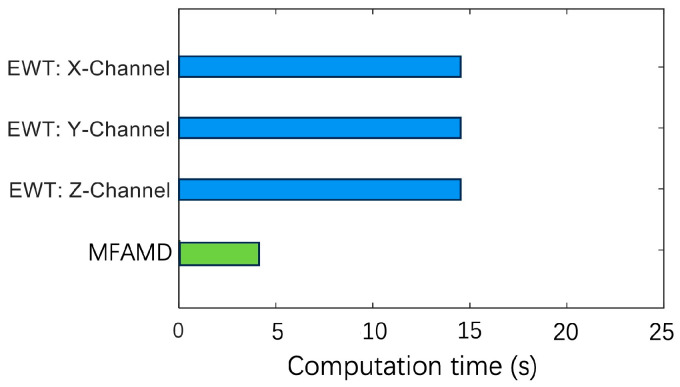
Comparison of computation time between EWT and MFAMD.

The number of boundaries segmented in the [2000–3000 Hz] frequency band cannot be used as a criterion for judging the excellence of the algorithm. This section measures the algorithm’s efficiency by dividing the frequency range across the 2500 Hz boundary by the total frequency band. Figure 13 is obtained based on the number and position of segmentation boundaries obtained by the two methods in Figure 9 and Figure 11. The figure shows that the MFAMD method suggested in this paper has a decomposition efficiency of 89%, while the EWT method’s efficiency for the three channel signals is less than 20%. The aforementioned assertion demonstrates the effectiveness of the MFAMD approach, which is suggested in this paper, in extracting fuse signals and fault information, showing that its segmentation approach makes more sense than the other two. Figure 14 presents the computation time for signal processing using the EWT and MFAMD methods. The computation time for the three-channel signal using the EWT method is approximately 15 s, and processing each channel separately requires around 45 s or even longer. The EWT method not only demands longer computation time but also yields less satisfactory results. In contrast, the proposed MFAMD method requires only about 4.8 s and achieves significantly better processing outcomes. The comparison of computation times further validates the feasibility of the proposed method.

## 4. Experimental Analysis

**Figure 15:**
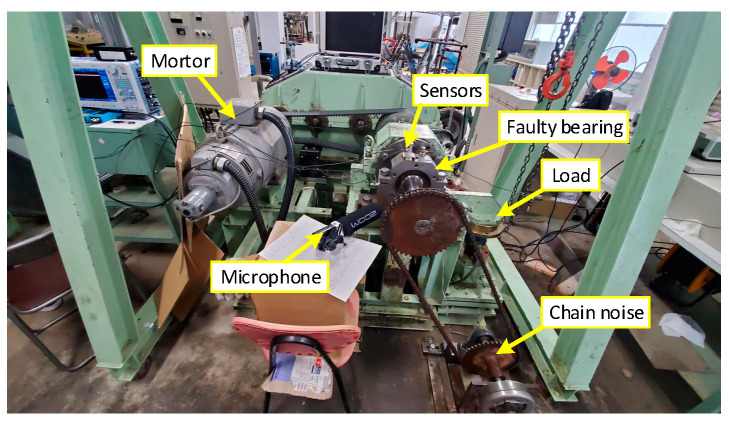
The test bench.

The Mie University test bench gathered the experimental data used in this section, as seen in Figure 15. Through the pulley, the shaft that connects the drive end and the bearing component rotates at a speed of 70 rpm. A rusty chain is used in the experiment to introduce random noise, which brings the bearing closer to its typical operation. The multi-channel sensor simultaneously gathers signals throughout the experiment. This paper uses the collected acoustic signal with a sampling frequency of 96 kHz, a sampling time of about 20 s, three collections, and an interval of 1 min between each collection to ensure the randomness of the noise.

### 4.1. Bearing Outer Ring

**Figure 16:**
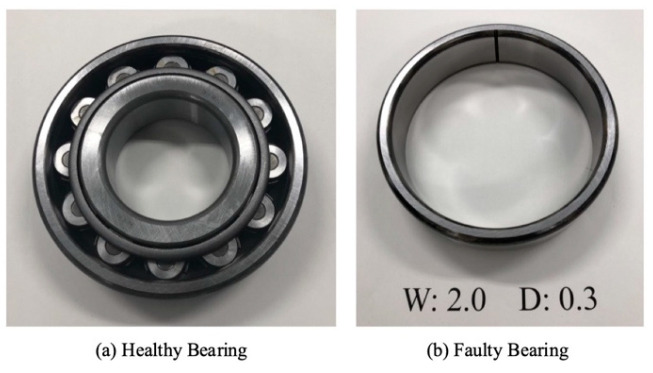
Healthy bearing and outer ring faulty bearing.

The rolling bearing model that we employed in the bearing outer ring failure experiment is NTN NU312. As illustrated in Figure 16, the bearing’s outer ring has been synthetically altered to exhibit linear damage that is 0.3 mm deep and 2.0 mm wide.

**Figure 17:**
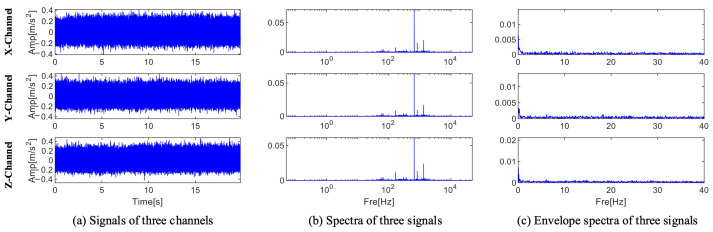
Time domain waveform, spectrum, and envelope spectrum of a bearing with outer ring.

The three sets of sound signals that the experimental platform gathered are each considered to be three channel signals. The time domain waveform, spectrum, and envelope spectrum for each channel are shown in Figure 17. The time domain waveform’s periodic pulses are drowned out by noise. The spectrum contains no specific fault information. Nevertheless, neither frequency doubling nor fault characteristic frequency are visible in the envelope spectrum. As a result, direct analysis cannot identify the issue with this set of signals.

**Figure 18:**
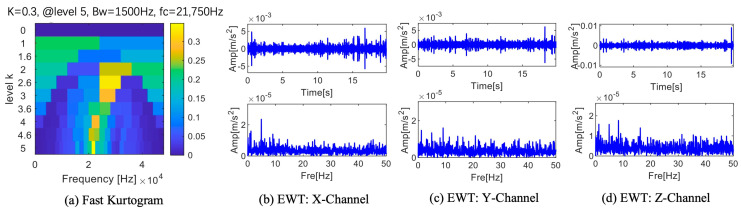
Results of fast kurtogram.

An excellent algorithm fast kurtogram was used to process this set of data. Since the effects of fast kurtogram on the three data sets are similar, only one set is shown in Figure 18a. As seen in Figure 18a, the fast kurtogram processing result reaches its maximum value in the fifth level, its bandwidth is 1500 Hz, and its center frequency is 21,750 Hz. The waveform and envelope spectrum of this frequency range are displayed in Figure 18b–d. The time domain waveform shows a single pulse but no periodicity. The fault frequency appears once in the Z channel’s envelope spectrum, but a direct and efficient fault diagnosis is impossible without the double frequency.

**Figure 19:**
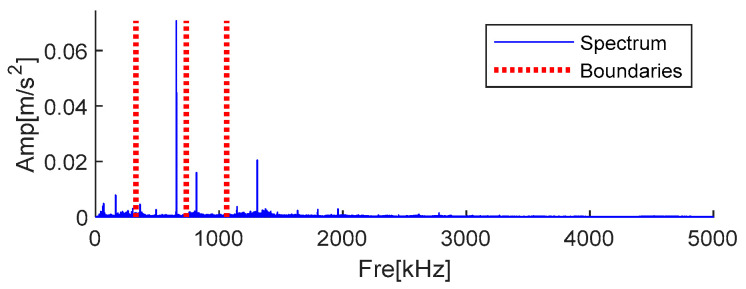
Bisecting frequencies obtained by AMD.

The traditional AMD method requires manual determination of important frequencies, and the bisection frequencies are calculated to distinguish different components. Because the three-channel signals are similar, the crucial amplitude information is most likely within 10 kHz, as seen in Figure 17b. By looking at the spectrum’s amplitude, as indicated in Table 2, one can determine the crucial frequencies. Since the three-channel signals are relatively similar, after calculation, the same boundary information is obtained because the signals are similar. The segmentation boundary is shown in Figure 19, and the horizontal axis is partially enlarged.

**Figure 20:**
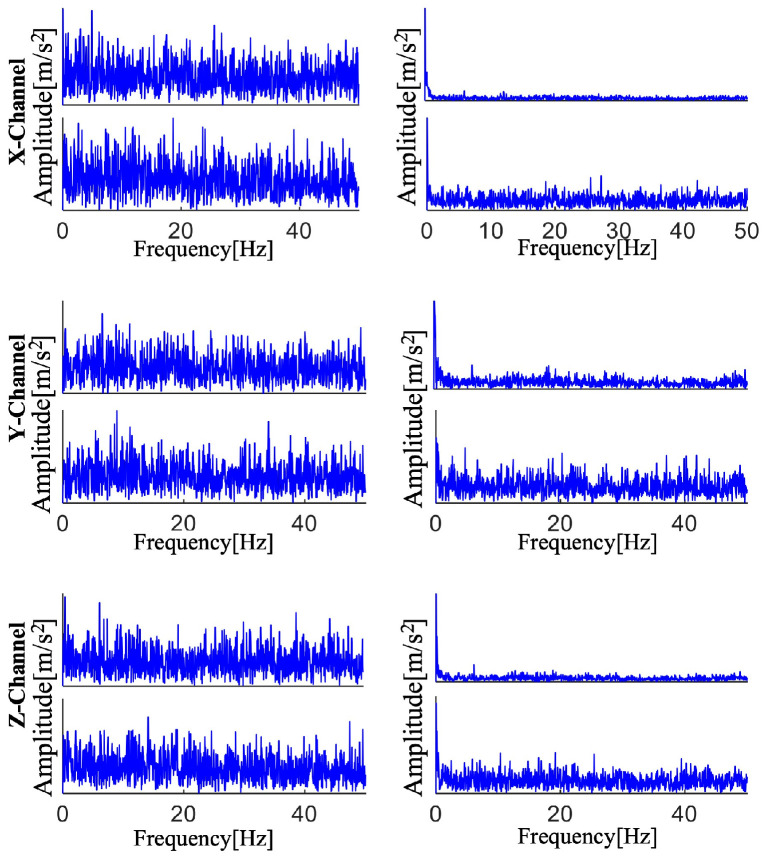
AMD’s breakdown of each component’s envelope spectrum.

In order to determine its envelope spectrum, each of the three channels’ four components is extracted and reconstructed into the time domain signal, as illustrated in Figure 20. Frequency multiples and the fault characteristic frequency are not visible in the envelope spectrum. This indicates that a new approach is required to replace this ineffective and unsuccessful strategy.

**Figure 21:**
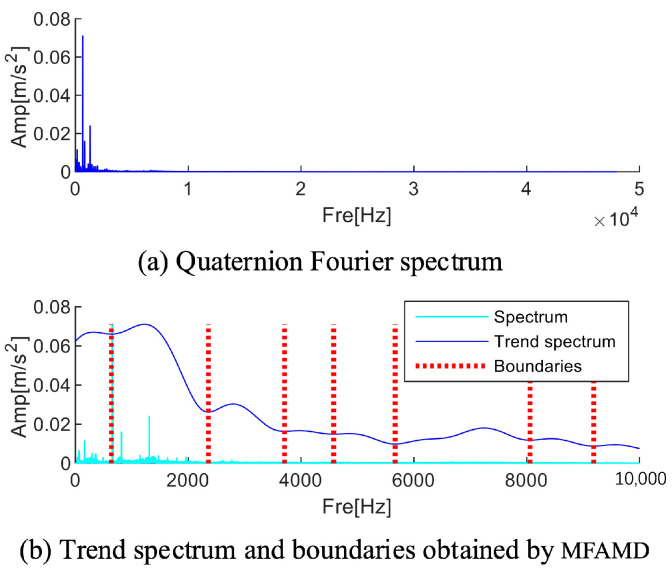
Results of segmentation using the multi-fusion analytic mode decomposition method.

The limitations of conventional AMD can be optimized by the MFAMD approach put forth in this paper. The MFAMD method first integrates the three-channel signals into quaternions. The frequency domain fusion method of different channel signals or multiple groups of signals can gather the advantages of each channel signal, which is more advantageous for the next step of processing. Figure 21a displays the spectrum following the frequency domain fusion of the three channel signals. The figure illustrates that the fused spectrum has more low-frequency amplitude information. As a result, as illustrated in Figure 21b, spectrum segmentation is carried out using the MFAMD method on the frequency band within 10 kHz, dividing it into eight components.

**Figure 22:**
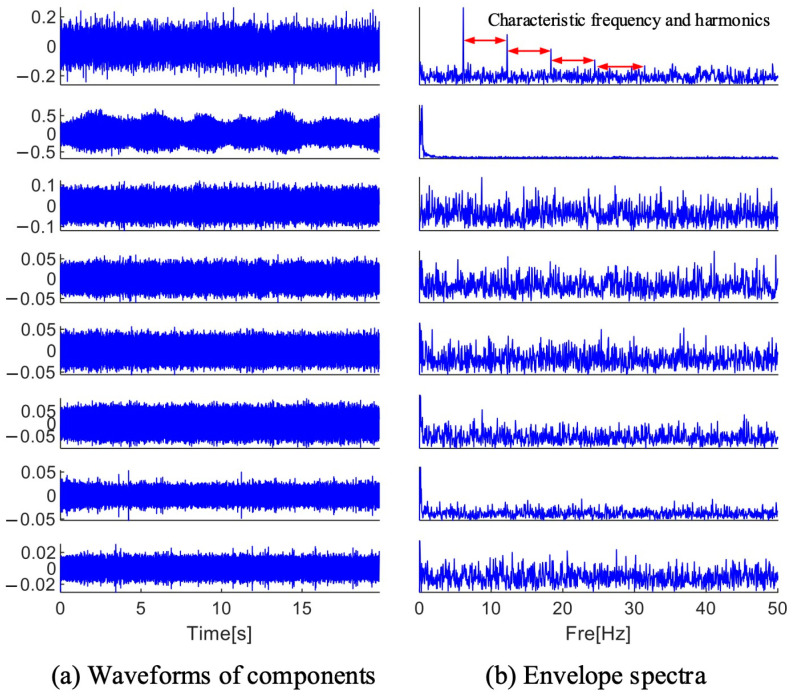
Results decomposed by MFAMD.

As shown in Figure 22, the envelope spectrum of each frequency band component is computed after it has been extracted and reconstructed into a time domain waveform. In the reconstructed time domain waveform, a large amount of noise is still found, and no periodic pulses are found, but throughout the first frequency band component’s reconstructed envelope spectrum, the fault characteristic frequency and its four times frequency are found, so that the bearing can be diagnosed to have a fault. The results show that the MFAMD method proposed in this paper can effectively diagnose the fault information in the acoustic signal.

### 4.2. Bearing Inner Ring

**Figure 23:**
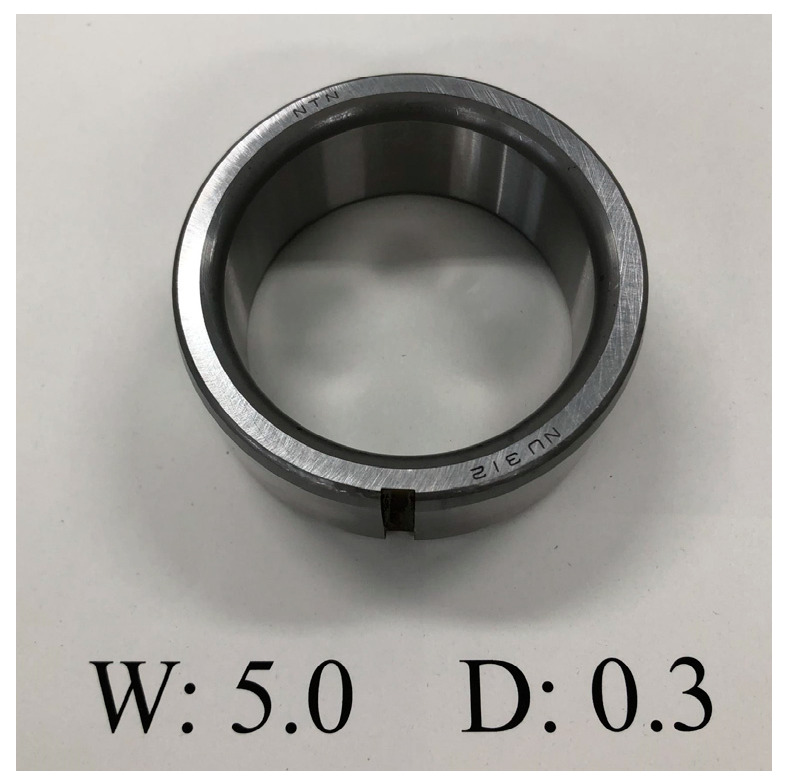
Faulty bearing.

**Figure 24:**
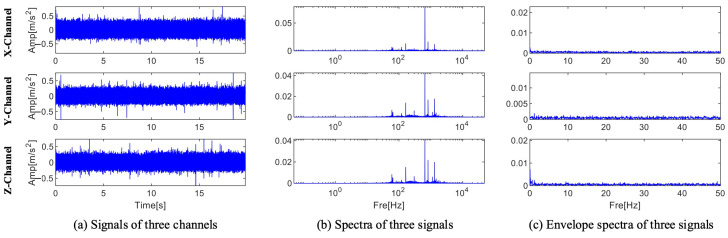
Time domain waveform, spectrum, and envelope spectrum of a bearing with inner ring.

As illustrated in Figure 23, the bearing’s inner ring has a crack brought on by artificial processing. The three-channel signal’s envelope spectrum, spectrum, and time domain waveform are displayed in Figure 24. As can be seen from the figure, fault diagnosis is not possible because the information with the larger amplitude in the spectrum is also concentrated within 10 kHz, and no fault frequency is seen in the envelope spectrum.

**Figure 25:**
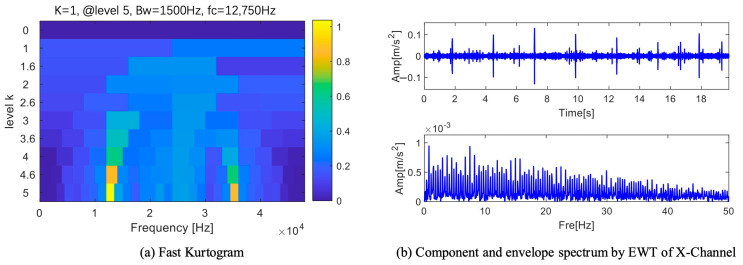
Results of fast kurtogram of X-channel.

**Figure 26:**
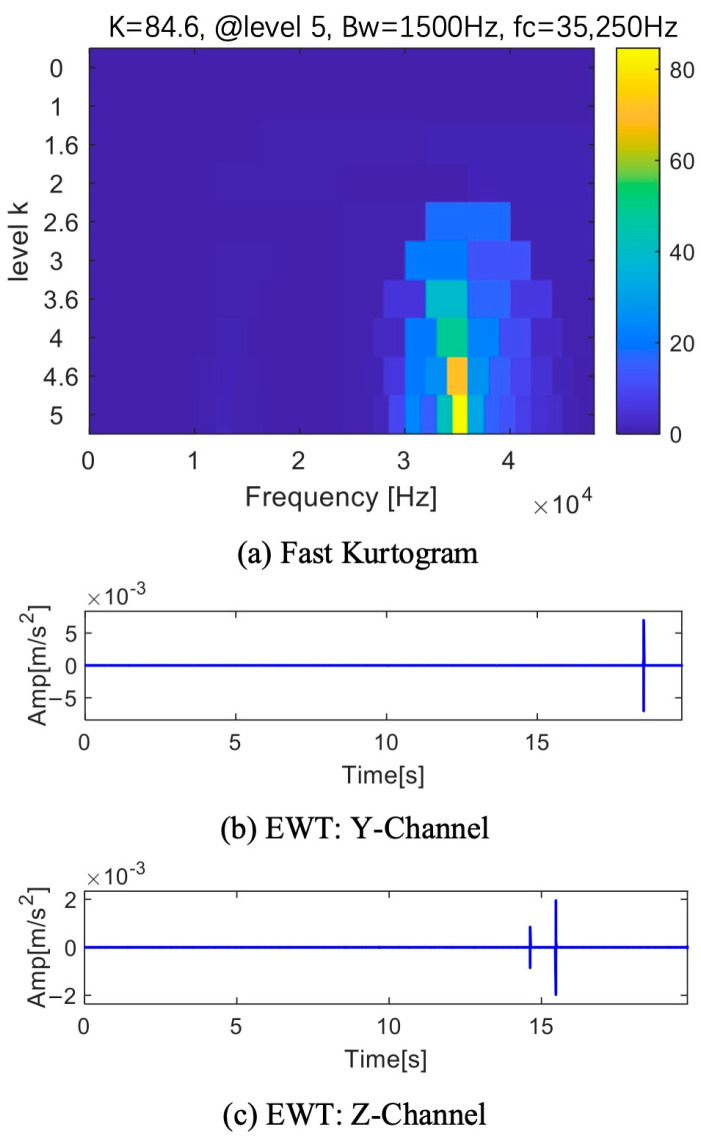
Fast kurtogram of Y or Z-channel results.

Figure 25a displays the outcome of processing the X-channel using fast kurtogram. With a bandwidth of 1500 Hz and a center frequency of 12,750 Hz, the fifth layer has the highest value computed. Figure 25b displays the envelope spectrum and time domain waveform that were acquired by removing this frequency band for reconstruction. Even though the time domain waveform shows periodic pulses, the characteristic frequency 0.374 Hz does not correspond to the fault frequency. The pulse period is not an accurate predictor of fault information since the fault frequency does not coincide with this frequency. Only one channel is displayed because the other two’s results are comparable, as seen in Figure 26a. The highest value is identified as the 24th frequency band component in the fifth layer, which has a center frequency of 35,250 Hz and a bandwidth of 1500 Hz. Figure 26b,c displays the time domain waveforms that were produced by reconstructing the frequency band components of the two channels. The time domain waveforms clearly show pulses, but they lack periodicity, making fault diagnosis impossible.

**Figure 27:**
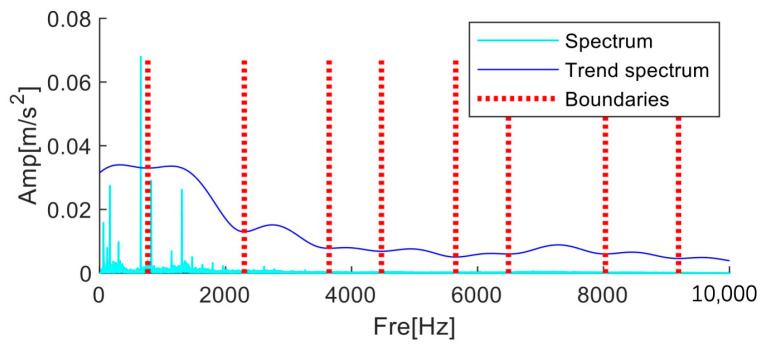
Trend spectrum and boundaries obtained by MFAMD.

**Figure 28:**
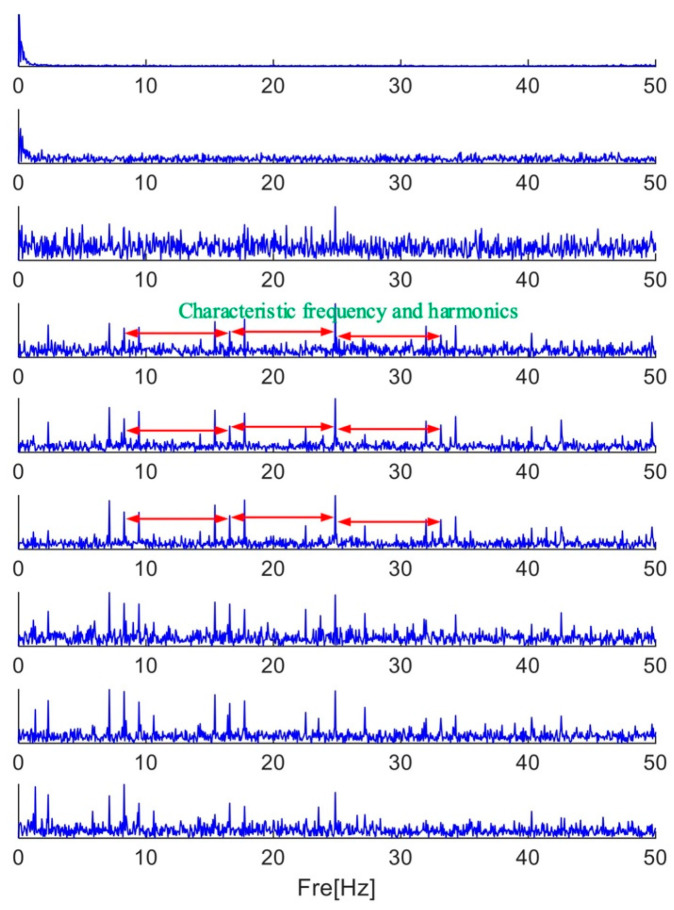
Results decomposed by MFAMD.

Figure 27 displays the outcome of processing the signal using the MFAMD method suggested in this paper. Nine parts make up the portion with a frequency below 10 kHz. As illustrated in Figure 28, the time domain signal is reconstructed, its envelope spectrum is computed, and each frequency band component is extracted independently. The envelope spectra of the fourth to eighth frequency band components show the inner ring fault characteristic frequency and its several frequencies. Thus, the bearing inner ring signal can be diagnosed using the MFAMD method in this paper, confirming the method’s efficacy.

## 5. Conclusions

This study proposes an MFAMD method to process multi-channel acoustic signals and effectively extract the hidden fault features. This method successfully expands the application scope of the traditional AMD method by fusing multiple groups or multi-channel signals in the frequency domain, enabling it to process multiple groups of long-time series acoustic signals in parallel. Secondly, after constructing the multi-channel acoustic signals into quaternion signals, the effective integration and feature enhancement of fault information are achieved. Finally, combined with the proposed quaternion Fourier spectrum trend segmentation algorithm, the best binary frequency can be automatically determined, multiple groups or multi-channel signals can be fused in the frequency domain, and the fused spectrum can be divided and modally decomposed. The simulation experimental results show that compared with the traditional method, the MFAMD method has significant advantages: while maintaining less boundary division, it can more effectively capture the sideband frequency impact components reflecting the fault characteristics and show the best decomposition efficiency. This feature gives it important application value in the field of mechanical fault diagnosis. In subsequent research, we will concentrate on investigating a method for parameter optimization that will allow adaptive parameter optimization for the algorithm, improving its generalizability and leading to precise and quick defect identification.

## Figures and Tables

**Table 1:** Number of boundaries around 2500 Hz and their effective impact.

Number	EWT X-Channel	EWT Y-Channel	EWT Z-Channel	FK Lv4	MFAMD
Total Boundaries	6	5	2	3	2
Effective impact	1	1	1	0	6

**Table 2:** Important frequencies of AMD for signals.

Channel	Important Frequencies (Hz)		
X	653.912	817.415	1307.870
Y	653.760	817.112	1307.520
Z	653.608	816.960	1307.170

## Data Availability

Data are contained within the article.
